# Subpathway-LNCE: Identify dysfunctional subpathways competitively regulated by lncRNAs through integrating lncRNA-mRNA expression profile and pathway topologies

**DOI:** 10.18632/oncotarget.12005

**Published:** 2016-09-13

**Authors:** Xinrui Shi, Yanjun Xu, Chunlong Zhang, Li Feng, Zeguo Sun, Junwei Han, Fei Su, Yunpeng Zhang, Chunquan Li, Xia Li

**Affiliations:** ^1^ College of Bioinformatics Science and Technology, Harbin Medical University, Harbin, 150081, China; ^2^ Department of Medical Informatics, Daqing Campus, Harbin Medical University, Daqing, 163319, China

**Keywords:** lncRNA, mRNA, cancer, pathway identification, competitive regulation

## Abstract

Recently, studies have reported that long noncoding RNAs (lncRNAs) can act as modulators of mRNAs through competitively binding to microRNAs (miRNAs) and have relevance to tumorigenesis as well as other diseases. Identify lncRNA competitively regulated subpathway not only can gain insight into the initiation and progression of disease, but also help for understanding the functional roles of lncRNAs in the disease context. Here, we present an effective method, Subpathway-LNCE, which was specifically designed to identify lncRNAs competitively regulated functions and the functional roles of these competitive regulation lncRNAs have not be well characterized in diseases. Moreover, the method integrated lncRNA-mRNA expression profile and pathway topologies. Using prostate cancer datasets and LUAD data sets, we confirmed the effectiveness of our method in identifying disease associated dysfunctional subpathway that regulated by lncRNAs. By analyzing kidney renal clear cell carcinoma related lncRNA competitively regulated subpathway network, we show that Subpathway-LNCE can help uncover disease key lncRNAs. Furthermore, we demonstrated that our method is reproducible and robust. Subpathway-LNCE provide a flexible tool to identify lncRNA competitively regulated signal subpathways underlying certain condition, and help to expound the functional roles of lncRNAs in various status. Subpathway-LNCE has been developed as an R package freely available at https://cran.rstudio.com/web/packages/SubpathwayLNCE/.

## INTRODUCTION

In recent years, with the development of next generation sequencing technologies, large scale long non-coding RNA (lncRNA) have been identified [1, 2]. It has been reported that lncRNA play crucial roles in various key biological processes [3–5], including post-transcriptional regulation [6], tumorigenesis and human disease [7, 8]. Increasing evidence indicated that lncRNAs can competitively regulate mRNAs expression levels by sharing common miRNA binding sites with mRNAs, which is an important widespread layer of RNA regulation [9, 10]. The first confirmation for ceRNA hypothesis in mammalian cells is about PTEN and its pseudogene [11]. PTENP1, which is a PTEN pseudogene and contains many seed matches for PTEN-targeting miRNAs, has been experimentally validated that it can act as a ceRNA for PTEN gene [11, 12]. The study of Wang et al. suggest that lincRNA-RoR may function as a endogenous miRNA sponge to regulate stemness factors (Oct4, Sox2, and Nanog) and then mediate the ESC maintenance and differentiation processes [13]. It has been demonstrated that H19 which is an oncogenic genes in multiple cancers, function as a competing endogenous RNA to inhibit miR-138 and miR-200a and further led to the de-repression of core genes for mesenchymal cells such as ZEB1/ZEB2 in colorectal cancer [14]. In addition, the study of Sumazin et al. proposed that the RNA-RNA competing interaction network could regulate oncogenic pathways in glioblastoma [15]. As the RNA competitive interaction can impact important functions in disease, identifying lncRNA competitively regulated pathways is thus not only can gain insight into the underlying mechanism and also help for exploring the functional roles of lncRNAs in disease. However, there are few methods can systematically predict dysfunctional pathways competitively regulated by lncRNAs under disease conditions.

Recently, several useful tools that investigate lncRNA function has been developed [16, 17]. For example, Linc2GO annotated lincRNA function based on the competing endogenous RNA hypothesis [16]. LncRNA2Function predicted lncRNA function based on their co-expression protein coding genes across 19 normal tissues [17]. However, currently, the function of lncRNAs underlying certain disease condition have not be well characterized. Additional strategy that explore lncRNA functional roles in the disease context is needed. In addition, Li *et al.* demonstrate that key local subregions, rather than completely pathways, is more subtly explainable to the etiology of diseases [18, 19]. It suggest that concentrating more attention on subpathways rather than entire pathways might be more meaningful in identification of disease-relevant pathway and explain the functional roles of lncRNAs in disease.

In this article, we proposed a novel method called Subpathway-LNCE, to identify lncRNAs competitively regulated signal subpathways underlying certain conditon. Firstly, KEGG signal pathways were converted into undirected graphs with genes as nodes and regulated relations as edges. Then, we reconstructed condition-specific lncRNA competitively regulated signal pathways (LRSP) based on matched lncRNA-mRNA expression profiles and their shared miRNAs. We mapped interesting lncRNAs and genes into LRSP, then located subpahtways within pathways according to the “lenient distance” similarity method [19]. Finally, the significance of candidate subathways was evaluated by using the Wallenius approximation [20]. In the result section, we firstly applied Supathway-LNCE on prostate data set and LUAD data set to demonstrate the effectiveness of our method. Then we analyzed kidney renal clear cell carcinoma(KIRC) data set to detect kidney cancer key competitively regulated lncRNAs that were biologically meaningful. Meanwhile, our results displayed reproducible by analyzing there independent prostate cancer data sets from different data sources. We also tested robust of our method by randomly disturbing matched expression profiles and LRSP.

## RESULTS

We evaluated Subpathway-LNCE method using prostate cancer data sets and KIRC data set. Firstly, we demonstrated the effectiveness of our method by identifying lncRNAs competitively regulated subpathways for prostate cancer. We then detected KIRC key competitively regulated lncRNAs that were biologically meaningful. Meanwhile, our results displayed reproducible by analyzing there independent prostate cancer data sets from different data sources. We also tested robust of our method by randomly disturbing matched expression profiles and LRSP.

### Identifying signal subpathways competitively regulated by LncRNAs for prostate cancer

We first applied Subpathway-LNCE with SRA data set of prostate cancer to assess the effectiveness [21]. Subpathway-LNCE identified 28 significant lncRNAs competitively regulating subpathways involved 26 complete pathways with FDR < 0.01 (Supplementary Table S1), of which up to 20 were reported to be associated with cancers, and well reported to be associated with tumor occurrence, development and metastasis (Supplementary Table S1). Furthermore, Subpathway-LNCE located key subregions which were more effective. It is obvious that Subpathway-LNCE can detect LncRNAs competitively regulated pathways that are biologically meaningful.

**Figure 1:**
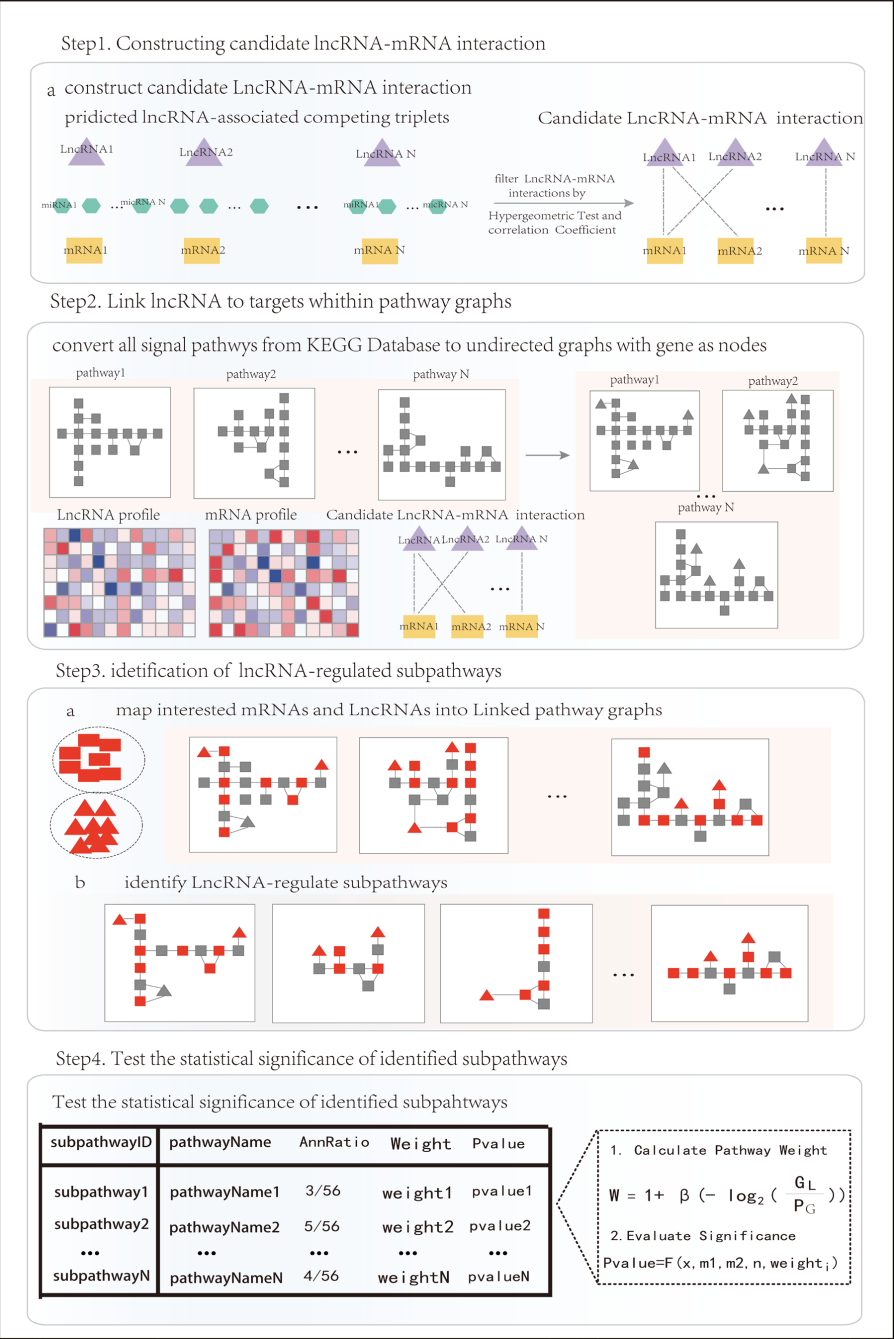
The pipeline overview of Subpathway-LNCE

In further analysis, we focused on three subpathways that competitively regulated by lncRNAs (Figure 1). The first is the most significant subpathway path: 04020_1, which was a subregion of calcium signaling pathway (Figure 1A, Supplementary Table S4). Calcium signaling as an intracellular messenger had been confirmed participate in many biological process, which had closely associated with cancers [22]. We then further explored this subpathway, and found that this subregion was competitively regulated by 8 lncRNAs. Among these lncRNAs, RP11-1398P2.1.1, which was a differential lncRNA, was compititive regulator of PPIF. Apoptosis regulator PPIF, deletion or reduction the expression levels of it could suppress cell proliferation and promote cell migration and invasion [23]. Down regulation of lncRNA MEG3 had been reported closely associated with several cancers, such as lung cancer, gastric cancer, etc [24–26]. Moreover, MEG3 played an important role in the molecular etiology of prostate cancer, which suggested the potential application of MEG3 in prostate cancer therapy [27]. The second significant subpathways was path: 04510_1, an important sub region within focal adhesion pathway (Figure 1B, Supplementary Table S5). In this subpathway, extracellular matrix (ECM) was the protein located in the upstream, which played an important role in different metastatic contexts in cancers [28, 29]. Notably, it was coordinately regulated by four lncRNAs, MIAT, MEG8, AC005682.5.1, and AC078937.4.1. Among these lncRNAs, MEG8 and AC078937.4.1 were differentially expressed. Another significant differently expressed lncRNA was LINC00087, which competitively regulated vascular endothelial growth factor (VEGF) and cyclin D2 (CCND2). Overexpression of the differential gene VEGF could promote angiogenesis and tumourogenesis in prostate cancer, and targeting the VEGF receptor pathway had shown promising early clinical application [30, 31]. Although CCND2 was a non-differential gene, LINC00087 which competitively regulated it were differentially expressed. CCND2 had an inhibitory potential on the proliferation of androgen receptor (AR)-dependent prostate cancer cells [32]. A high Cyclin D2 methylation levels was related with clinicopathologic features of tumor aggressiveness in prostate cancer [33]. LINC00087 that competitively regulated VEGF and CCND2 suggested that it may play an important role in prostate cancer. Additional, DLEU2 and HOTAIRM1 were involved in this subpathway. These two lncRNAs had been reported play important role in the leukemia cells [34–36]. In addition, DLEU2 was the host gene of miR-15a and miR-16-1 which were important tumor suppressors [37]. DLEU2 and HOTAIRM1 competitively regulated PPP1CC and ROCK1 respectively. It was notable that downstream of PPP1CC and ROCK1 was MYL9, which was a famous hallmark tumor gene [38]. Moreover, it had been reported that MYL9 may efficiently predict recurrence-free survivals in prostate cancer patients [39]. In summary, these result suggest that our method can not only identify biological meaningful subpathways, but also highlight some critical lncRNAs underlying disease condition.

The third subpathway, path: 04070_1, was a part of Phosphatidylinositol signaling system (Figure 1C, Supplementary Table S6). In this subpathway, phosphatase and tensin homologue deleted on chromosome-10 (PTEN) was a tumor suppressor gene that was competitively regulated by three LncRNAs, FDG5-AS1, WDFY3-AS2 and CTD -2302E22.2.1. PTEN inactivation had a correlation with many different types of cancer including prostate cancer [40, 41]. Moreover, PTEN negatively regulated activity of the PI3K/Akt/mTOR pathway, which played a prominent role in prostate tumor development [42, 43]. We then further focused on the underlying miRNAs that shared by PTEN and these three lncRNAs. We found that most miRNAs were associated with tumor. For example, among them miR-214 was shared by PTEN and all three lncRNAs of competitive regulators, was reported to be diagnostic potential biomarker in prostate cancer urine specimens with qRT-PCR experiment [44]. Mir-17 family were known as oncomiRs and had essential functions for tumorigenesis [45]. In particular, by targeting miR-17-5p and miR-106a-5p, PTEN expreesion were rescued resulting in reducing tumor growth *in vivo* of prostate cancer [45]. Though functions of FDG5-AS1, WDFY3-AS2 and CTD -2302E22.2.1 were unclear, Subpathway-LNCE reveals that they may play important role in prostate cancer by competing cancer related miRNAs with PTEN to disturb the phosphatidylinositol signaling system.

### Identifying signal subpathways competitively regulated by LncRNAs for LUAD dataset

To examine the utility of Subpathway-LNCE for diseases, we applied Subpathway-LNCE with TCGA data set of LUAD. Subpathway-LNCE identified 28 significant lncRNAs competitively regulated subpathways in LUAD data sets with FDR<0.01 (Supplementary Table S3), of which up to 15 were well reported to be associated with tumor occurrence, development and metastasis (Supplementary Table S3). It was note that Small cell lung cancer was on the top rank in the Supathway-LNCE result list. In this subregion, HCG-18 competitively regulated gene CCND1, which was an oncogene acted as a driver of multiple types of human malignancies [46, 47]. CCND1 dysregulation was associated with cellular proliferation and tumor growth of lung tumor [48]. Furthermore, Subpathway-LNCE located key subregions which were more effective. It is obvious that Subpathway-LNCE can detect lncRNAs competitively regulated pathways that are biologically meaningful.

### Dissecting key lncRNAs in KIRC-relevant lncRNA competitively regulated signal subpathways network

**Figure 2:**
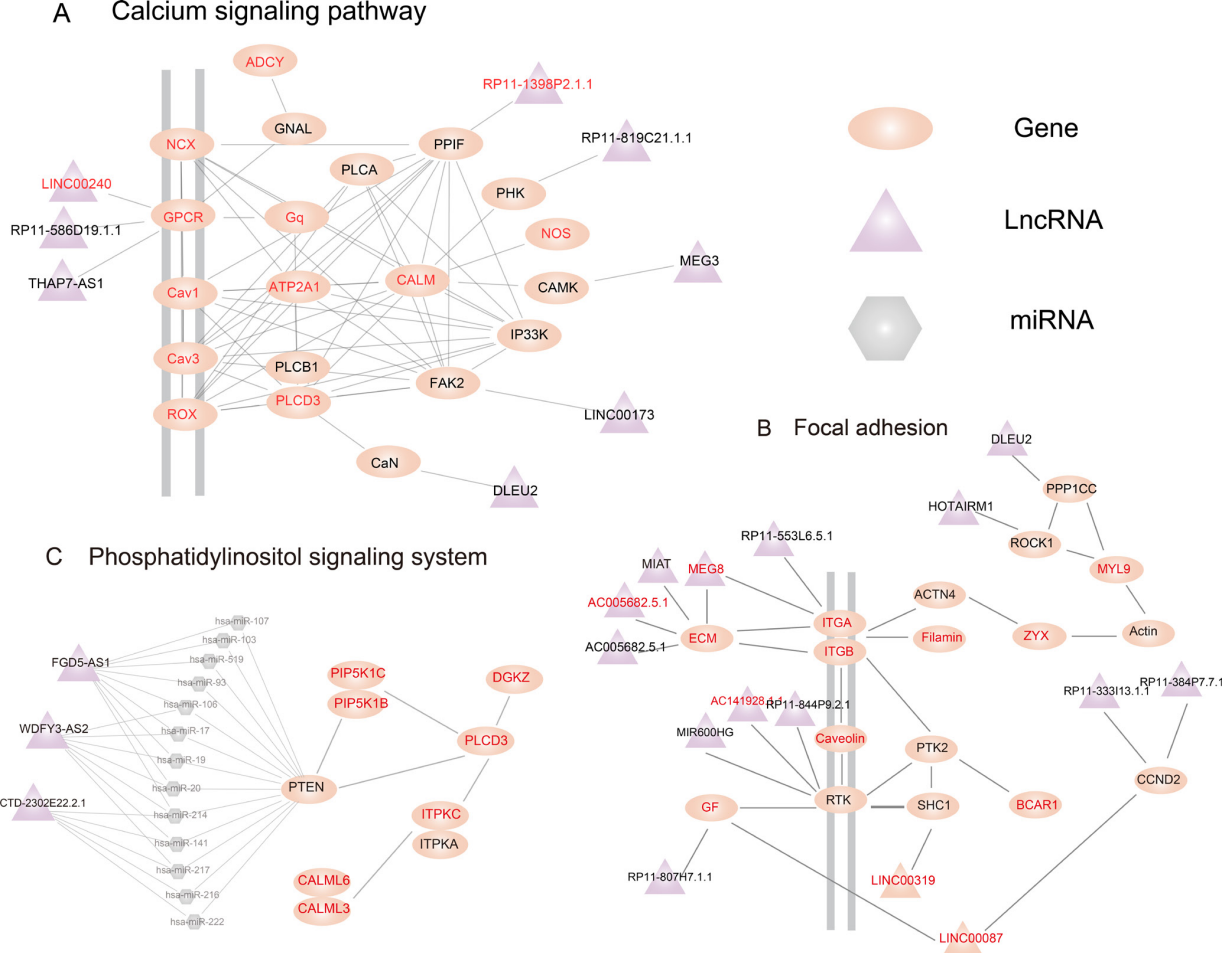
Subpathways identified using the subpathway-LNCE method Ellipse, triangle and hexagon nodes represent genes, lncRNAs and miRNAs, respectively. Red and balck node labels represent differential and non-differential genes/lncRNAs, respectively, and gray node labels represent miRNAs. (**A**) Calcium signaling subpathway (path: 04020_1, *FDR*= 7.87E-14). (**B**) Focal adhesion subpathway (path: 04070_1, *FDR*= 7.19E-12). (**C**) Phosphatidylinositol signaling system subpathway (path:00030_1, *FDR*=0.20E-2).

In this section, we focused on applying our method to dissect key lncRNAs that implicated with disease and further explore its functional roles under disease condition. To do this, we performed Subpathway-LNCE on KIRC dataset. Firstly, we constructed the KIRC-relevant lncRNA competitively regulated signal subpathways network, in which the top rank 20 subpathways in Supathway-LNCE result list and lncRNA that competitively regulated these subpathways were considered. These 20 subpathways belonging to 19 complete pathways of which up to 14 had been well reported associated with cancers (Supplementary Table S2). Hub nodes are always very important in the biological network as the connectivities of which are extremely high. We thus focused on hub lncRNAs in the lncRNA competitively regulated signal subpathways network. We selected the top 10% of lncRNAs with the highest degrees in the lncRNA-subpathway network as the hub lncRNAs. We then merging 20 subpathways including relevant mRNAs and lncRNAs to construct KIRC relevant modulatory network. Finally, we obtained the KIRC relevant modulatory network involved 19 pathways, 14 hub LncRNAs and 48 gene/proteins (Figure 2A).

The modulatory relationship HCG18- EGFR appeared up to 10 pathways, of which 7 pathways were cancer-related such as focal adhesion, such as MAPK signaling pathway and etc. EGFR was a receptor tyrosine kinase, its signaling ultimately increased angiogenesis and decreases apoptosis [45]. Overexpressed gene EGFR was a famous driver gene in mass of human tumors and had well be used as targeted therapy [49, 50]. Furthermore, it had experiments demonstrated dysregulation of the EGFR pathway was associated with growth and invasion in cancer [51]. HCG-18 competitively regulated another gene CCND1, which was an oncogene acted as a driver of multiple types of human malignancies [46, 47]. CCND1 dysregulation was associated with cellular proliferation and tumor growth of kidney tumor [52]. Another differential hub lncRNA PVT1 was also competitively regulated CCND1 and some other cancer related genes to disturb cancer hallmark pathways including p53 signaling pathway. It had been reported that PVT1 was associated with multiple types of human malignancies, including prostate cancer, pancreatic ductal adenocarcinoma, ovarian cancer etc [37, 53–55]. In addition, the hub lncRNA DLEU2 competitively regulated pathway up to 14 pathways. It was a critical host gene of miR-15a and miR-16-1 which inhibited tumorigenicity both *in vitro* and *in vivo* and frequently deleted in malignancy [37]. The above result suggest that these hub lncRNAs such as HCG-18, DLUE2 and PVT1 may play key roles in the initiation and progression of kidney cancer.

To further detect key lncRNAs of competitive regulation in the KIRC relevant modulatory network, we applied K-mean clustering method for survival analysis on hub lncRNAs in the network. The kidney cancer samples were divided into two groups basing on the expression quantity of the corresponding lncRNAs with *p* value = 0.0378 (Figure 2C), related Gene ID and sample ID of Figure 2B were in Supplementary Files (Supplementary Table S6). Meanwhile, each hub lncRNA have been applied for survival analysis separately (Supplementary Figure S4, Supplementary Table S3). Then, two groups of patients including high expression and low expression group were divided based on the mean value of the expression quantity of each hub lncRNA. The results showed that most hub lncRNA can't divide two groups very well for survival analysis separately. It suggested that these hub lncRNAs may play a coordinately regulated role in KIRC. We have applied survival analysis for TCGA data set of prostate cancer, as the same method with KIRC dataset. We applied K-mean clustering method for survival analysis on hub lncRNAs in the PRAD regulated network. The prostate cancer samples were divided into two groups basing on the expression quantity of hub lncRNAs in the network with *p value* = 0.0118 (Supplementary Figure S5). Furthermore, we performed the survival analysis for each hub lncRNA separately. Firstly, we divided the patients into two groups based on the mean value of the expression quantity of each hub lncRNA. Then, we applied survival analysis for the two groups of patients. As a result, all the hub lncRNA can't divide two groups very well for survival analysis separately (Supplementary Figure S6). It suggested that these hub lncRNAs may play a coordinately regulated role, the results showed consistency with the KIRC data set. These indicate that expression of these lncRNAs were associated with kidney cancer patient survival. Furthermore, most of hub lncRNAs exhibit high expression in the high risk group comparing with that in the low risk group (Figure 2B). The above result provided further evidence for the potential key roles of hub lncRNAs in kidney cancer.

### Reproducibility and robustness analyses

#### Reproducibility analysis

**Figure 3:**
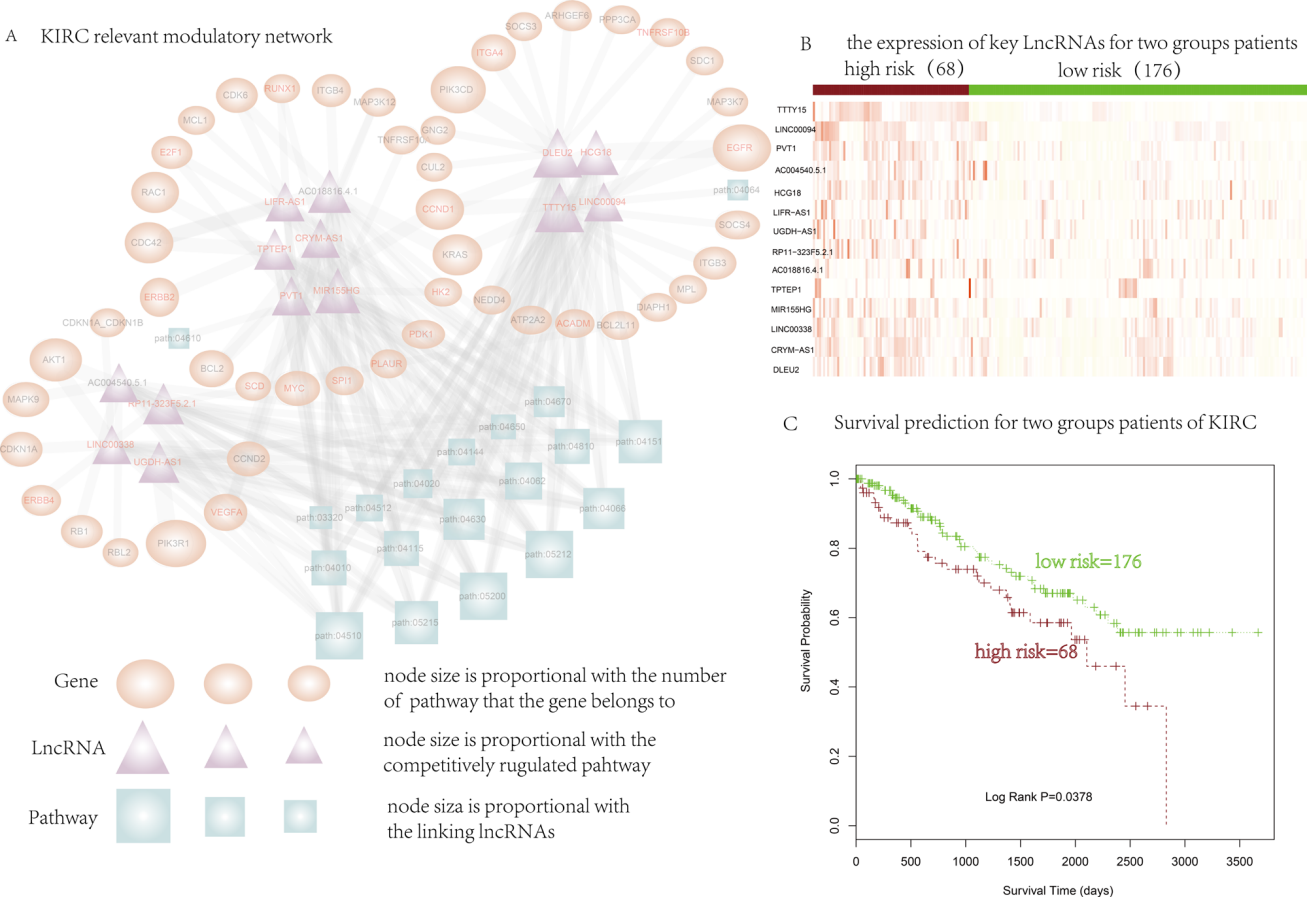
(**A**) KIRC-relevant lncRNA competitively regulated signal subpathways network. Circle and triangle nodes represent genes and lncRNAs, respectively, and square represent subpathway. The lncRNA/subpathway node size is proportional to the number of directly interacted subpathways/lncRNAs, and the gene node size is proportional to the number of appeared subpathways. The edge width of lncRNA-gene pair is proportional to the number of subpathways they involved in. (**B**) The expression quantity of key LncRNAs (rows) for two groups patients (colums). (**C**) Kaplan-Meier survival analysis of two groups of patients with different clinical outcomes. Survival days are shown along the X axis. Overall survival rates are shown along the Y axis.

In this section, we aim to evaluated the reproduciblity of Subpathway-LNCE, we applied Subpathway-LNCE with three independent prostate cancer data sets from three different resource, including Sequence Read Archive (SRA), TCGA Data Portal (TCGA) and Gene Expression Omnibus (GEO), details see methods. We focused on pathways that rank on top 15 of the result list from these three different data sets. Hypergeometric test was used to evaluate the significance of shared pathways between any two data sets from different resource. We found that pathways shared between SRA and TCGA with *p value* = 5.66e-10, and the pathways up to 80% were associated with cancers. Hypergeometric test of shared pathways between TCGA and GEO under a threshold of *p* = 8.56e-7, whereas pathways shared between SRA and GEO with *p value*= 1.84e-5 (Figure 3). Futhermore, the number of pathways shared among SRA, GEO and TCGA data sets result was up to 6, and outstandingly, all shared pathways had been well reported associated with cancers. We further restriction to top 10 pathways in the result list (Supplementary Figure S2), the results were above all, though three independent prostate the data sets was from three different resource. We have further analyzed the results of subpathway-LNCE based on different *P value* of differential expression gene (0.05, 0.1, 0.2) in the same data sets for all three different data resources. And we focused on pathways that rank on top 15 of the result list from different *P value* of differential expression gene in the same data sets for all three different data sets. The number of pathways shared under different *P value* was up to 10 in the TCGA data set, and in the other data set the number also up to 8 and 7, respectively (Supplementary Figure S7). In any two different *P value*, the hypergeometric test of shared pathways was significant. All above indicated that the results obtained using Subpathway-LNCE were reproducible.

#### Robustness analysis

We used two methods to test the stability of Subpathway-LNCE, namely randomly introducing noise in the matched mRNA-lncRNA expression profiles and disturbing LRSP. We firstly randomly deleted mRNAs and lncRNAs from mached mRNA-lncRNA expression profiles, and deleted percentage from 5% to 30%, at 5% intervals. Then we repeated the deleted process 100 times and applied the Subpathway-LNCE method on noisily matched mRNA-lncRNA expression profiles. We calculated the mean ratio of recall significant pathways comparing to original significant pathways (FDR < 0.01). In general, the deleted percentage increased, the overlap of significant pathways fell slowly. The Subpathway-LNCE shown the best stability when the deleted ratio was 5%, recalling more than 90% of the pathways. Even after removal of up to 15% of the expression data, the recalling still more than 70%. It indicated that Subpathway-LNCE was robust when the expression data was noise.

We next randomly deleted the edges within LRSPs from 5% to 30%, at 5% intervals, then we repeated the deleted process 100 times and applied the Subpathway-LNCE method on SRA prostate data sets for each disturbed LRSP. The mean ratio of recall significant pathways was calculated comparing to original significant pathways (FDR < 0.01). We found that as the deleted percentage increased, the overlap of significant pathways fell slowly (Figure 3), and the recalling still more than 60% even after disturbing up to 25% edges of LRSPs. Furthermore, Robustness analysis of the KIRC dataset was consistent with the above result (Supplementary Figure S1). Above all, it suggested that Subpathway-LNCE method was robust in resisting the disturbance of expression data and LRSPs.

## DISCUSSION

Recently, lncRNAs have been found can function as competitors of mRNAs for miRNA binding, thereby competitively regulating mRNA expression levels and maintain normal biological functions [9, 10, 14]. This regulatory mechanism may help understand biological problems and organism complexity. Disturbances of these lncRNA competitively regulated functions may led to diseases, but on the other hand, a better understanding this regulation may offer opportunities for new therapies. However, to the best of our current knowledge, there are few methods specifically designed to identify lncRNAs competitively regulated functions and the functional roles of these competitive regulation lncRNAs have not be well characterized in diseases. In this study, we proposed a novel method called Subpathway-LNCE, to identify lncRNAs competitively regulated signal subpathways underlying certain conditon, providing a powerful tool for exploring the regulation function of lncRNAs in human disease.

Subpathway-LNCE which integrated lncRNA-mRNA expression profile and pathway topologies was specifically designed to identify lncRNAs competitively regulated functions. It considers several important aspects as follows. Firstly, lncRNA, which play important roles in various biological processes [5, 6], represent a new regulatory layer and should be included in the pathway analysis. Second, when locating candidate subregions we take advantage of pathway topologies along with lncRNAs competitively regulated genes embedded in different pathways, which was better to reflect the transmission of disease signals. LncRNAs have sponge features which can competitively regulated biological pathways and thus play critical roles in the initiation and progression of diseases such as tumor [14], it is thus necessary to integrative analyze the joint effect of genes and lncRNAs that competitively regulated them by considering pathway topologies. Third, we have adopted a strategy of subpathway, rather than completely pathways, is more subtly explainable to the etiology of diseases. Moreover, concentrating more attention on subpathways rather than entire pathways might be recall and identify more biologically meaningful pathways and dissect the functional roles of lncRNAs. The input data of Subpathway-LNCE method needs matched lncRNA-mRNA expression profiles, Subpathway-LNCE provide a flexible tool to identify lncRNA competitively regulated signal subpathways underlying certain condition, and help to expound the functional roles of lncRNAs in various status.

In the result section, we applied Subpathway-LNCE with SRA data set of prostate cancer and choose three different sub region from different aspects to elaborate the effective of our methods. The first is the significant subpathway was path:04020_1, which was a subregion of calcium signaling pathway. Down regulation of lncRNA MEG3 had been reported closely associated with several cancers [25, 26]. Moreover, MEG3 played an important role in the molecular etiology of prostate cancer, which suggested the potential application of MEG3 in prostate cancer therapy [27]. The second was path: 04510_1, an important sub region within focal adhesion pathway. We focused the upstream protein of this sub region, which were more important because it influenced other genes/proteins downstream. Interestingly, the regulator of the upstream protein was four lncRNAs, which may play an important role for coordinated regulation and function. The third subpathway, path: 04070_1, was a part of Phosphatidylinositol signaling system. In this subpathway, we concerned the miRNAs behind PTEN and lncRNAs which was regulator of PTEN, and most miRNAs were associated with cancers. Meanwhile, We have supplied the other information of miRNA for the other two subpathways in Supplementary Files. (Supplementary Tables S4, S5). To further explore key LncRNAs of competitive regulation concerted to multiple subpathways, we constructed KIRC relevant modulatory network. In the KIRC relevant modulatory network, the modulatory relationship HCG18- EGFR appeared up to 10 pathways, of which 7 pathways were cancer-related such as focal adhesion, such as MAPK signaling pathway and etc. TCGA data set include miRNA expression data, we further analyzed the expression relationship between lncRNA, miRNA and mRNA. The miR-146a-5p was the micRNA shared by HCG18- EGFR, we found that the expression relationship between both HCG18-EGFR and miR-146a-5p was significantly negative. Moverer, miR-146a-5p have differential effects on growth and metastatization on cancer [56]. The differential hub lncRNA PVT1 was also competitively regulated CCND1 and some other cancer related genes to disturb cancer hallmark pathways including p53 signaling pathway. It had been reported that PVT1 was associated with multiple types of human malignancies, including prostate cancer, pancreatic ductal adenocarcinoma, ovarian cancer etc [53–55]. We found that PVT1 was the target of miR-106a-5p and miR-20b-5p, and the expression relationship between PVT1 and both miRNAs were significantly negative. Then we applied K-mean clustering method for survival analysis on hub lncRNAs in the network to detected key LncRNAs of competitive regulation in the KIRC relevant modulatory network. The kidney cancer samples were divided into two groups based on the expression value of the corresponding lncRNAs with log rank *p value* = 0.0378. It suggested LncRNA may be responsible for explaining disease processes thereby presenting opportunities for new therapies.

In this study, there are differences among three data sets, including experimental method, operational approach etc. So we considered false discovery rate (FDR) to identify differentially expressed genes in different data sets. Actually, many researchers took different threshold values among different data sets for identifying differential genes. For example, Liang et al. applied different FDRs in their study [57]. We then further explored the fold change of these differential genes in each dataset, the mean value of fold change among three different data sets all >= 1.5 (Supplementary Figure S3). We used Pearson Correlation Coefficient to evaluate co-expression for any pair of relations in the candidate lncRNA-mRNA network based on matched lncRNA and mRNA expression profiles. And there are some convenient tools can evaluate co-expression. However, any other suitable method, such as Weighted Gene Co-Expression Network Analysis [58], can be used to calculate this co-expression coefficient.

In order to ensure the reliability of data, the mRNA-miRNA interactions were experimentally validated, collected from TarBase [59], mirTarBase [60], mir2Disease [61], miRecords (V4.0) [62]. The lncRNA-miRNA interactions were predicted using TargetScan (v.6.0) [63], PITA (March 2007 version) [64], miRanda (Nov. 2010 version) [65] and RNAhybrid (v.2.1.1) [66] with default parameters. Then we have integrated the AGO-CLIP-seq data set into the pipeline to identify miRNA-binding sites on lncRNA sequences which have experimentally supported. By integrating genome coordinates of CLIP-seq peaks and predicted miRNA-binding sites, the reliability of data was further ensured. We have used published scientific documentation confirming the results from Subpathway-LNCE. For example, in the KIRC relevant modulatory network, the differential hub lncRNA PVT1 was also competitively regulated CCND1 and some other cancer related genes to disturb cancer hallmark pathways including p53 signaling pathway. It had been reported that PVT1 was associated with multiple types of human malignancies, including prostate cancer, pancreatic ductal adenocarcinoma, ovarian cancer etc [53–55]. The hub lncRNA DLEU2 was a critical host gene of miR-15a and miR-16-1 which inhibited tumorigenicity both *in vitro* and *in vivo* and frequently deleted in malignancy [37]. Although we didn't use experiments to confirm the results, we have supplied specific binding sites about lncRNA-miRNA data to guide a further experiment to verify the predicted results for researchers (Supplementary Table S7).

In summary, Subpathway-LNCE, a novel method that designed to identify lncRNAs competitively regulated signal subpathways, can not only help to understanding the molecular mechanism of diseases, but also will lead to important insight into the functional roles of lncRNAs in pathological states. Moreover, we have implemented Subpathway-LNCE method as an R-based package, which is publicly available on https://cran.rstudio.com/web/packages/SubpathwayLNCE/, and our tool provided a flexible usage for changing the significant positive threshold of *r* value.

## MATERIALS AND METHODS

### Materials

#### Prostate cancer datasets

We analyzed three independent prostate data sets, the three prostate data sets was obtained from different data sources: Sequence Read Archive (SRA, SRP002628) [21], The Cancer Genome Atlas dataset (TCGA, https://tcga-data.nci.nih.gov/tcga/) and Gene Expression Omnibus (GEO, GSE23316) [67], and each of them included cancer samples and normal samples.

(i) SRA data set of prostate cancer

To obtain transcription level of lncRNAs and mRNAs, firstly we used TopHat mapping reads to the reference genome (hg19) [68], next we estimated the relative abundances of each transcript using Cufflinks with transcript annotation file [69]. Thus, we obtained RPKM lncRNA and mRNA expression profiles. Whereas, to get read counts expression profiles, RNA-Seq reads were mapped to the reference genome (hg19) using Tophat [68], then expression level was extracted using easyRNAseq method [70]. We used DESeq method to identify differentially expressed mRNAs based on read count expression profies [71]. The matched lncRNA and mRNA expression profiles, which included 10 normal samples and 20 cancer samples. MRNAs and lncRNAs were considered differentially expressed under a threshold of FDR = 0.1.

(ii) TCGA data set of prostate cancer

The PRAD data set was download from TCGA, and corresponding clinical information was also obtained. The matched mRNA/lncRNA expression profile for prostate cancer were extracted according to our previous study [72]. In brief, RNA-seqV2 data were downloaded from TCGA level 3 data sets, and RPKM (Reads Per Kilobase per Million mapped reads) values for lncRNA/mRNA were recalculated using the following method: RPKM = (raw read count × 10^9)/(total reads), where raw read counts = sum of raw read counts in all exons mapped entirely within the lncRNA/mRNA locus; total reads = sum of raw read counts calculated for all exons of a single sample; = sum of length of exons mapped the LncRNA / mRNA locus. Annotation of exons mapping to lncRNA/mRNA was extracted from GENCODE (V19). Finally, the matched lncRNA and mRNA expression profiles included 494 cancer samples and 42 normal samples. We used DESeq method to identify differentially expressed mRNAs based on read count expression profiles [71]. A mRNA was considered to be differentially expressed with false discovery rate (FDR) < 1e-4.

(iii) GEO data set of prostate cancer

We mapped 604 258 probe sequences (25 bp in length) to annotated files from GENCODE (GRCH37) with BLAST under probe re-annotation pipeline (see Supplementary Files), resulted with 17254 mRNAs and 3495 lncRNAs. We obtained matched lncRNA and mRNA expression profiles, which included 6 normal samples and 12 cancer samples. MRNAs were considered differentially expressed under a threshold of FDR = 0.1 of Student's *t* test.

#### KIRC datasets

The KIRC data set was download from TCGA, and corresponding clinical information was also obtained. Details of these data sets were processed similar with TCGA data set of prostate cancer, which has described above. And the matched lncRNA and mRNA expression profiles included 71 normal samples and 255 cancer samples.

#### LUAD datasets

The LUAD data set was download from TCGA. Details of the data-set were processed similar with TCGA data set of prostate cancer, which has described above. And the matched lncRNA and mRNA expression profiles included 52 normal samples and 494 cancer samples.

#### Methods

**Figure 4:**
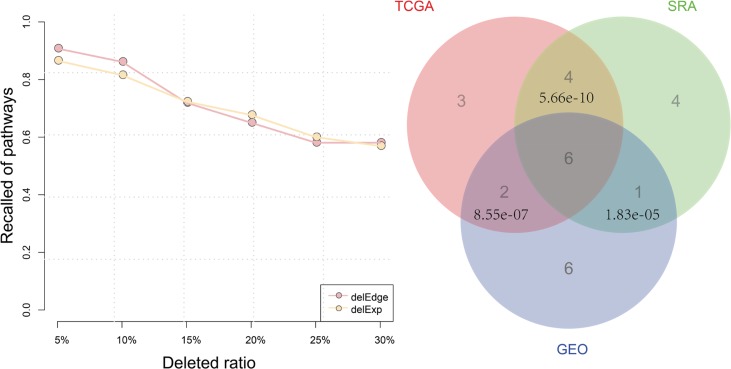
Reproducibility and robustness analyses (**A**) Robustness analysis. Pink line shows the mean ratio of recalled pathways using Subpathway-LNCE method for prostate cancer data set after randomly deleting *N*% of genes and miRNAs from the corresponding profiles, where *N* = 5, 10, …, 30. Light green line shows the mean ratio of for prostate cancer data set recalled pathways using Subpathway-LNCE after randomly deleting *N*% of the edges in each RMPG, where *N* = 5, 10, …, 30. (**B**) Reproducibility analysis. Venn diagram depicts top 15 pathways identified by Subpathway-LNCE in three independent prostate cancer data sets from three different resource, including Sequence Read Archive (SRA), TCGA Data Portal (TCGA) and Gene Expression Omnibus (GEO).

Subpathway-LNCE was developed to identify lncRNA competitively regulated signal subpathways underlying certain conditon. The schematic overview is shown in Figure 4. It includes four main parts: (i) Firstly, we constructed candidate lncRNA-mRNA competitively regulated network; (ii) KEGG signal pathways were converted into undirected graphs with genes as nodes and regulated relations as edges, we reconstructed condition-specific lncRNA competitively regulated signal pathways (LRSP) based on matched lncRNA and mRNA expression profiles and lncRNA-mRNA competitively regulated network; (iii) We mapped competing lncRNAs and interesting genes into LRSP, then locates subpahtways within pathways according to the “lenient distance” similarity method; (iv) We evaluated the significance of candidate subpathways by using the Wallenius approximation. The details are displayed below.

#### Constructing candidate lncRNA-mRNA interaction

Firstly, we collected lncRNA-associated competing triplets (lncRNA-miRNA-mRNA relationships). The lncRNA-miRNA interactions were obtainted from our previously work and StarBase, whereas experimentally validated mRNA-miRNA interactions were collected from TarBase [59], mirTarBase [60], mir2Disease [61], miRecords (V4.0) [62]. Then, we constructed candidate LncRNA-mRNA competitively regulated relationships based on their shared miRNAs. For each lncRNA, we identify its candidate competing mRNAs as follows: (i) hypergeometric test of shared miRNAs under a threshold of *p* = 0.05 (ii) Jaccard Coefficient of shared miRNAs rank at top 20%. In order to ensure the reliability of data, those relationships which satisfied with both criteria were retained. Finally, we obtained candidate LncRNA-mRNA competitively regulated network included 6722 lncRNA-mRNA interactions among 1527 genes and 798 LncRNAs.

#### Reconstructing condition-specific LncRNA-regulated signal pathways

##### Linking LncRNAs to regulated-mRNAs within pathway graphs

We converted 191 KEGG signal pathways into undirected graphs kept original pathway structural information using our previously developed R packages [19]. We used Pearson Correlation Coefficient to evaluate co-expression for any pair of relations in the candidate lncRNA-mRNA network based on matched lncRNA and mRNA expression profiles, those *r* value had reached a significant positive threshold were retained (*p <* 0.05) based on Fisher's Z transform [73]. Then, these lncRNAs were embedded into pathway graphs as nodes by linking to their regulated-mRNAs. Finally, we obtained condition-specific lncRNA competitively regulated signal pathways (LRSP), which included lncRNA nodes and lncRNA-mRNA competitively regulated edges.

##### Locate subpathways competing regulated by lncRNAs

LncRNAs involved in the competing regulation and genes of interests were regarded as signature nodes. These nodes combined with topology of LRSP can help us efficiently positioning lncRNA-regulated subregions. We first mapped signatures nodes into LRSP, then locating subpathways competing regulated by lncRNAs used “lenient distance” similarity combined with network topology structure. In brief, we calculated the shortest path between any two signature nodes, if the number of molecules between each signature pairs was no longer than n, then they were merged into one nodes. Finally, the number of nodes in the molecule sets within pathway no less than s were regarded as candidate subpahtways competing regulated by LncRNAs. The *n* and *s* parameters control the intensity of regulated signals and the size of candidate subpathways, respectively. We used *n = 1* and *s* = 8 as default parameters.

##### Evaluated the significance of candidate subathways

To estimate whether the candidate subpathways were competing regulated by lncRNAs comparing random, we used the Wallenius approximation methods to estimate the significance of candidate subpathways. The following parameters were needed: (i) the number of interesting mRNAs (x); (ii) the number of background mRNAs (n); (iii) the number of background mRNAs involved in this subpathways (m1); (iv) the number of interesting mRNAs annotated into this subpathway (m2); (v) the weight of this subpathway (w), which suggested the intensity of competing regulation by lncRNAs involved in this subpathways. The weight of the subpathway was computed as follows:

In above formula, parameter P_G_ is the number of mRNAs of this subpathways, and parameter G_L_ is the number of mRNAs competitively regulated by lncRNAs within this subpathway. β is parameter of control (In this study β = 1). The Wallenius approximation methods was executed using R package BiasedUrn [20].

$$W=1+\beta \left(-\log_{2}\left(\frac{G_{L}}{P_{G}}\right)\right)$$

##### Survival analysis

K-mean clustering was used to classify KIRC (PRAD) patients into two groups by expression of hub lncRNAs in the lncRNA competitively regulated subpathway network. Then, the Kaplan-Meier method was used to evaluate the difference of survival time between these two groups, and statistical significance was estimated by log-rank test.

## SUPPLEMENTARY MATERIALS






