# Supplementary material for: Subpathway-LNCE: Identify dysfunctional subpathways competitively regulated by lncRNAs through integrating lncRNA-mRNA expression profile and pathway topologies
Source: Oncotarget. 2016 Sep 13;7(43):69857–70. doi: 10.18632/oncotarget.12005 (PMC5342520; doi:10.18632/oncotarget.12005)
Supplement: Supplementary file 1 [file oncotarget-07-69857-s001.pdf]

# Subpathway-LNCE: Identify dysfunctional subpathways competitively regulated by lncRNAs through integrating lncRNA-mRNA expression profile and pathway topologies

Supplementary Materials

## SUPPLEMENTARY TEXT

### workflow of ReAnnotation

The 25-nt probe sequence was downloaded from the manufacturers' website (http://www.affymetrix.com). Then they are mapped to the sequences of protein-coding transcripts and long non-coding RNA transcripts from GENCODE[1] project using BLASTn. After that, a serious of filtering process was applied to the mapping result:

i) We only kept the probes which are perfectly matched to the reference sequences and classified them into two sets, correlating with protein-coding transcripts and long non-coding RNA transcripts respectively.

ii) Probes which mapped to protein-coding transcripts and long non-coding RNA transcripts simultaneously were removed.

iii) All of the remaining transcripts are then mapped to gene level for further analysis.

iv) Probes mapped to different genes are removed.

v) Only genes mapped with at least three probes are retained.

vi) According to the probe-gene mapping relationships, a new environment variable is created by R and can be used together with Affy packages to generate expression files of protein-coding genes and long non-coding RNAs.

## REFERENCES

1. Harrow J, Frankish A, Gonzalez JM, Tapanari E, Diekhans M, Kokocinski F, Aken BL, Barrell D, Zadissa A, Searle S, Barnes I, Bignell A, Boychenko V et al. GENCODE: the reference human genome annotation for The ENCODE Project. Genome Res. 2012; 22:1760–1774.
2. Tennakoon S, Aggarwal A, Kallay E. The calcium-sensing receptor and the hallmarks of cancer. Biochim Biophys Acta. 2015.
3. Zhao F, Huang W, Zhang Z, Mao L, Han Y, Yan J, Lei M. Triptolide induces protective autophagy through activation of the CaMKKbeta-AMPK signaling pathway in prostate cancer cells. Oncotarget. 2016; 7:5366–5382. doi: 10.18632/oncotarget.6783.
4. Chatterji T, Varkaris AS, Parikh NU, Song JH, Cheng CJ, Schweppe RE, Alexander S, Davis JW, Troncoso P, Friedl P, Kuang J, Lin SH, Gallick GE. Yes-mediated phosphorylation of focal adhesion kinase at tyrosine 861 increases metastatic potential of prostate cancer cells. Oncotarget. 2015; 6:10175–10194. doi: 10.18632/oncotarget.3391.
5. Hensley PJ, Desiniotis A, Wang C, Stromberg A, Chen CS, Kyprianou N. Novel pharmacologic targeting of tight junctions and focal adhesions in prostate cancer cells. PLoS One. 2014; 9:e86238.
6. Liu P, Cheng H, Roberts TM, Zhao JJ. Targeting the phosphoinositide 3-kinase pathway in cancer. Nat Rev Drug Discov. 2009; 8:627–644.
7. Rivas-Fuentes S, Salgado-Aguayo A, Pertuz Belloso S, Gorocica Rosete P, Alvarado-Vasquez N, Aquino-Jarquin G. Role of Chemokines in Non-Small Cell Lung Cancer: Angiogenesis and Inflammation. J Cancer. 2015; 6:938–952.
8. Engelman JA. Targeting PI3K signalling in cancer: opportunities, challenges and limitations. Nat Rev Cancer. 2009; 9:550–562.
9. Insua-Rodriguez J, Oskarsson T. The extracellular matrix in breast cancer. Adv Drug Deliv Rev 2015.
10. Wagner EF, Nebreda AR. Signal integration by JNK and p38 MAPK pathways in cancer development. Nat Rev Cancer. 2009; 9:537–549.
11. Masoud GN, Li W. HIF-1alpha pathway: role, regulation and intervention for cancer therapy. Acta Pharm Sin B. 2015; 5:378–389.
12. Mesnil M, Yamasaki H. Bystander effect in herpes simplex virus-thymidine kinase/ganciclovir cancer gene therapy: role of gap-junctional intercellular communication. Cancer Res. 2000; 60:3989–3999.
13. Song H, Hollstein M, Xu Y. p53 gain-of-function cancer mutants induce genetic instability by inactivating ATM. Nat Cell Biol. 2007; 9:573–580.
14. Larue L, Bellacosa A. Epithelial-mesenchymal transition in development and cancer: role of phosphatidylinositol 3' kinase/AKT pathways. Oncogene 2005, 24:7443–7454.
15. Yamaguchi H, Condeelis J. Regulation of the actin cytoskeleton in cancer cell migration and invasion. Biochim Biophys Acta. 2007; 1773:642–652.
16. Ricci A, Greco S, Mariotta S, Felici L, Bronzetti E, Cavazzana A, Cardillo G, Amenta F, Bisetti A, Barbolini G.

Neurotrophins and neurotrophin receptors in human lung cancer. Am J Respir Cell Mol Biol. 2001; 25:439–446.

17. Ben-Neriah Y, Karin M. Inflammation meets cancer, with NF-kappaB as the matchmaker. Nat Immunol. 2011; 12:715–723.

18. Yu H, Rohan T. Role of the insulin-like growth factor family in cancer development and progression. J Natl Cancer Inst. 2000; 92:1472–1489.

19. Segawa Y, Yoshimura R, Hase T, Nakatani T, Wada S, Kawahito Y, Kishimoto T, Sano H. Expression of peroxisome proliferator-activated receptor (PPAR) in human prostate cancer. Prostate. 2002; 51:108–116.

20. Wallace PK, Howell AL, Fanger MW. Role of Fc gamma receptors in cancer and infectious disease. J Leukoc Biol. 1994; 55:816–826.

21. Boudny V, Kovarik J. JAK/STAT signaling pathways and cancer. Janus kinases/signal transducers and activators of transcription. Neoplasma. 2002; 49:349–355.

22. Kagi D, Ledermann B, Burki K, Seiler P, Odermatt B, Olsen KJ, Podack ER, Zinkernagel RM, Hengartner H. Cytotoxicity mediated by T cells and natural killer cells is greatly impaired in perforin-deficient mice. Nature. 1994; 369:31–37.

23. Restifo NP, Esquivel F, Kawakami Y, Yewdell JW, Mule JJ, Rosenberg SA, Bennink JR. Identification of human cancers deficient in antigen processing. J Exp Med. 1993; 177:265–272.

24. He J, Baum LG. Endothelial cell expression of galectin-1 induced by prostate cancer cells inhibits T-cell transendothelial migration. Lab Invest. 2006; 86:578–590.

25. Jafari S, Saeidnia S, Abdollahi M. Role of natural phenolic compounds in cancer chemoprevention via regulation of the cell cycle. Curr Pharm Biotechnol. 2014; 15:409–421.

26. Appert-Collin A, Hubert P, Cremel G, Bennasroune A. Role of ErbB Receptors in Cancer Cell Migration and Invasion. Front Pharmacol. 2015; 6:283.

27. Elgui de Oliveira D, Muller-Coan BG, Pagano JS. Viral Carcinogenesis Beyond Malignant Transformation: EBV in the Progression of Human Cancers. Trends Microbiol. 2016.

28. Hu H, Luo ML, Desmedt C, Nabavi S, Yadegarynia S, Hong A, Konstantinopoulos PA, Gabrielson E, Hines-Boykin R, Pihan G, Yuan X, Sotirious C, Dittmer DP et al. Epstein-Barr Virus Infection of Mammary Epithelial Cells Promotes Malignant Transformation. EBioMedicine 2016.

**Supplementary Table S1: Subpathways identified using Subpathway-LNCE in prostate data set**

| pathwayId | pathwayName | Reference |
|---|---|---|
| path:04020_1 | Calcium signaling pathway | [2, 3] |
| path:04510_1 | Focal adhesion | [4, 5] |
| path:05200_1 | Pathways in cancer | [6] |
| path:04062_1 | Chemokine signaling pathway | [7] |
| path:04151_7 | PI3K-Akt signaling pathway | [8] |
| path:04512_1 | ECM-receptor interaction | [9] |
| path:04010_2 | MAPK signaling pathway | [10] |
| path:04066_1 | HIF-1 signaling pathway | [11] |
| path:04540_1 | Gap junction | [12] |
| path:04916_1 | Melanogenesis | |
| path:04115_1 | p53 signaling pathway | [13] |
| path:04151_1 | PI3K-Akt signaling pathway | [8] |
| path:04070_1 | Phosphatidylinositol signaling system | [14] |
| path:04810_3 | Regulation of actin cytoskeleton | [15] |
| path:05166_3 | HTLV-I infection | |
| path:04713_1 | Circadian entrainment | |
| path:04728_1 | Dopaminergic synapse | |
| path:04722_1 | Neurotrophin signaling pathway | [16] |
| path:04064_2 | NF-kappa B signaling pathway | [17] |
| path:05222_1 | Small cell lung cancer | |
| path:04141_2 | Protein processing in endoplasmic reticulum | |
| path:04725_1 | Cholinergic synapse | |
| path:04810_2 | Regulation of actin cytoskeleton | [15] |
| path:04910_2 | Insulin signaling pathway | [18] |
| path:05132_1 | Salmonella infection | |
| path:03320_1 | PPAR signaling pathway | [19] |
| path:04724_1 | Glutamatergic synapse | |
| path:04666_2 | Fc gamma R-mediated phagocytosis | [20] |

**Supplementary Table S2: Subpathways identified using Subpathway-LNCE in KIRC data set**

| pathwayId | pathwayName | Reference |
|---|---|---|
| path:04151_2 | PI3K-Akt signaling pathway | [8] |
| path:04510_1 | Focal adhesion | [4,5] |
| path:05200_2 | Pathways in cancer | [6] |
| path:04066_1 | HIF-1 signaling pathway | [11] |
| path:04512_1 | ECM-receptor interaction | [9] |
| path:04020_1 | Calcium signaling pathway | [3] |
| path:04062_1 | Chemokine signaling pathway | [7] |
| path:04610_1 | Complement and coagulation cascades | |
| path:04010_1 | MAPK signaling pathway | [10] |
| path:03320_1 | PPAR signaling pathway | [19] |
| path:04630_1 | Jak-STAT signaling pathway | [21] |
| path:04650_1 | Natural killer cell mediated cytotoxicity | [22] |
| path:04810_2 | Regulation of actin cytoskeleton | [15] |
| path:05212_1 | Pancreatic cancer | |
| path:05215_1 | Prostate cancer | |
| path:04144_4 | Endocytosis | |
| path:04612_4 | Antigen processing and presentation | [23] |
| path:04115_1 | p53 signaling pathway | [13] |
| path:04064_1 | NF-kappa B signaling pathway | [17] |
| path:04670_2 | Leukocyte transendothelial migration | [24] |

## Supplementary Table S3: Subpathways identified using Subpathway-LNCE in LUAD data set

| pathwayId | pathwayName | |
|---|---|---|
| path:05200_1 | Pathways in cancer | [6] |
| path:04151_1 | PI3K-Akt signaling pathway | [8] |
| path:04110_1 | Cell cycle | [25] |
| path:05166_1 | HTLV-I infection | |
| path:04510_1 | Focal adhesion | [4, 5] |
| path:05222_1 | Small cell lung cancer | |
| path:05161_1 | Hepatitis B | |
| path:04630_1 | Jak-STAT signaling pathway | [21] |
| path:04115_1 | p53 signaling pathway | [13] |
| path:04512_1 | ECM-receptor interaction | [9] |
| path:05212_1 | Pancreatic cancer | |
| path:04062_1 | Chemokine signaling pathway | [7] |
| path:05220_2 | Chronic myeloid leukemia | |
| path:05214_1 | Glioma | |
| path:04012_1 | ErbB signaling pathway | [26] |
| path:05203_18 | Viral carcinogenesis | [27] |
| path:05215_1 | Prostate cancer | |
| path:05223_1 | Non-small cell lung cancer | |
| path:03013_1 | RNA transport | |
| path:05169_6 | Epstein-Barr virus infection | [28] |
| path:05034_7 | Alcoholism | |
| path:05219_6 | Bladder cancer | |
| path:04066_1 | HIF-1 signaling pathway | [11] |
| path:04380_1 | Osteoclast differentiation | |
| path:04020_1 | Calcium signaling pathway | [3] |
| path:03320_1 | PPAR signaling pathway | [19] |
| path:04070_1 | Phosphatidylinositol signaling system | [14] |
| path:05218_2 | Melanoma | |

**Supplementary Table S4: Informations of miRNA for calcium signaling pathway**

| lncName | geneName | interSetMic |
|---|---|---|
| RP11-586D19.1 | CCKBR | hsa-miR-148a;hsa-miR-148b |
| RP11-819C21.1 | PHKB | hsa-miR-16 |
| RP11-1398P2.1 | PPIF | hsa-miR-21 |
| MEG3 | CAMK2G | hsa-miR-219a-5p |
| DLEU2 | PPP3CA | hsa-miR-30a-5p |
| LINC00240 | AGTR1 | hsa-miR-155-5p |
| ERVK13-1 | CHP1 | hsa-miR-16-5p |
| PDXDC2P | CHP1 | hsa-miR-16-5p |
| THAP7-AS1 | HTR2C | hsa-miR-22-3p |
| LINC00173 | PTK2B | hsa-miR-23b-3p |

**Supplementary Table S5: Sinformations of miRNA for focal adhesion pathway**

| lncName | geneName | interSetMic |
|---|---|---|
| RP11-333I13.1 | CCND2 | hsa-let-7a;hsa-let-7b;hsa-miR-106b; hsa-miR-15a;hsa-miR-17;hsa-miR-20a |
| RP11-384P7.7 | CCND2 | hsa-let-7a;hsa-let-7b |
| AC078937.4 | THBS1 | hsa-let-7a;hsa-let-7b;hsa-miR-92a;hsa-miR-98 |
| AC005682.5 | THBS1 | hsa-let-7a;hsa-let-7b;hsa-miR-98 |
| RP11-844P9.2 | MET | hsa-miR-199b-3p;hsa-miR-23b |
| RP11-807H7.1 | VEGFA | hsa-miR-106a;hsa-miR-106b;hsa-miR-147; hsa-miR-15b;hsa-miR-17;hsa-miR-20a;hsa-miR-20b; hsa-miR-330-3p;hsa-miR-34b;hsa-miR-520g; hsa-miR-520h;hsa-miR-93 |
| AC141928.1 | ERBB2 | hsa-miR-125a-5p;hsa-miR-125b |
| RP11-553L6.5 | ITGA2 | hsa-miR-16;hsa-miR-30a |
| LINC00087 | CCND2 | hsa-miR-106b-5p;hsa-miR-17-5p;hsa-miR-20a-5p |
| LINC00087 | VEGFA | hsa-miR-106a-5p;hsa-miR-106b-5p;hsa-miR-17-5p; hsa-miR-20a-5p;hsa-miR-20b-5p;hsa-miR-93-5p |
| MIR600HG | ERBB2 | hsa-miR-199a-5p;hsa-miR-199b-5p |
| LINC00319 | SHC1 | hsa-miR-141-3p;hsa-miR-200a-3p |
| HOTAIRM1 | ROCK1 | hsa-miR-148b-3p |
| MEG8 | ITGA2 | hsa-miR-16-5p |
| MEG8 | LAMB1 | hsa-miR-16-5p |
| MIAT | COL4A1 | hsa-miR-29a-3p;hsa-miR-29b-3p; hsa-miR-29c-3p |
| DLEU2 | PPP1CC | hsa-miR-30a-5p |

**Supplementary Table S6: Include Gene ID and sample ID of Figure 3B.**
See Supplementary_Table_S6
**Supplementary Table S7: Include information of lncRNA-mRNA binding sites.**
See Supplementary _Table_S7

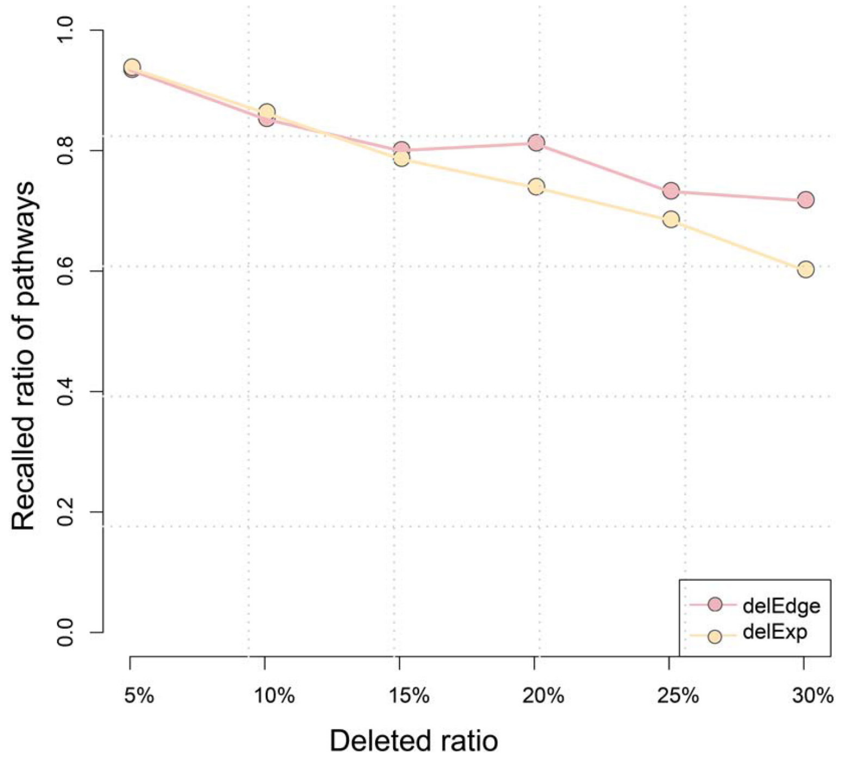


**Supplementary Figure S1: Pink line shows the mean ratio of recalled pathways using Subpathway-LNCE method for KIRC data set after randomly deleting *N%* of genes and miRNAs from the corresponding profiles, where *N* = 5, 10, ...,** 30. Light green line shows the mean ratio of for KIRC data set recalled pathways using Subpathway-LNCE after randomly deleting *N*% of the edges in each RMPG, where *N* = 5, 10, ..., 30.

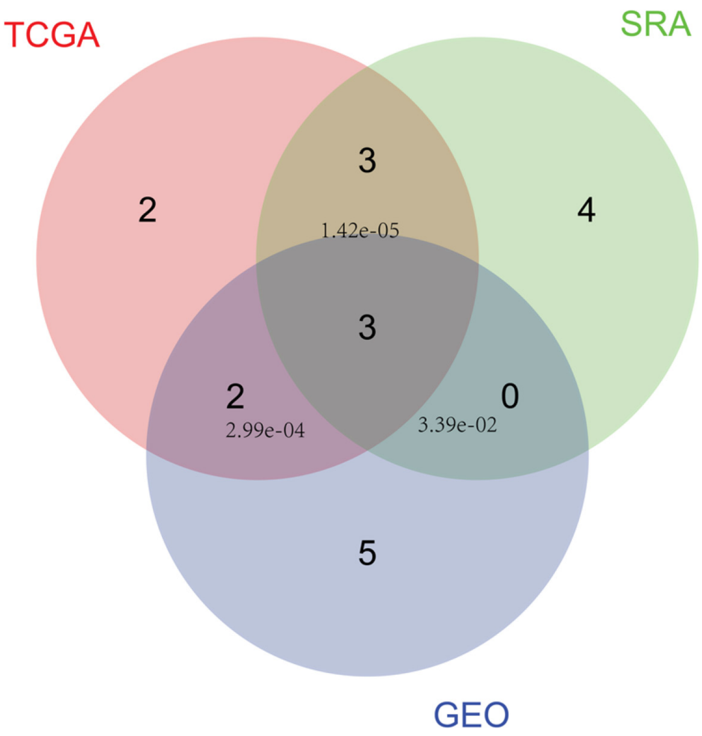


**Supplementary Figure S2: Reproducibility analysis.** Venn diagram depicts top 10 pathways identified by Subpathway-LNCE in three independent prostate cancer data sets from three different resource, including Sequence Read Archive (SRA), TCGA Data Portal (TCGA) and Gene Expression Omnibus (GEO).

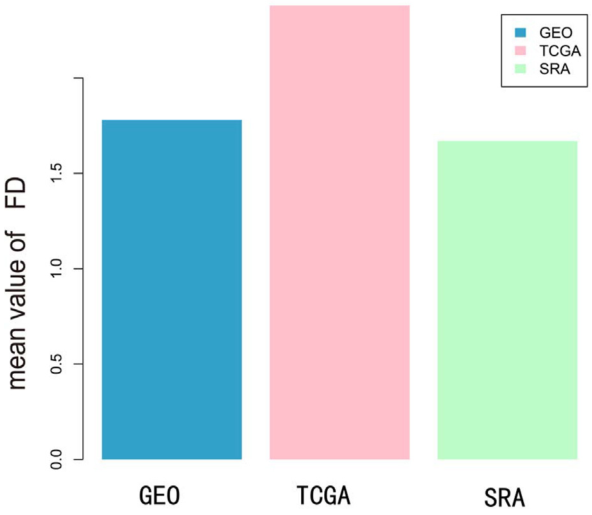


**Supplementary Figure S3: The mean value of fold change among three different data sets.**

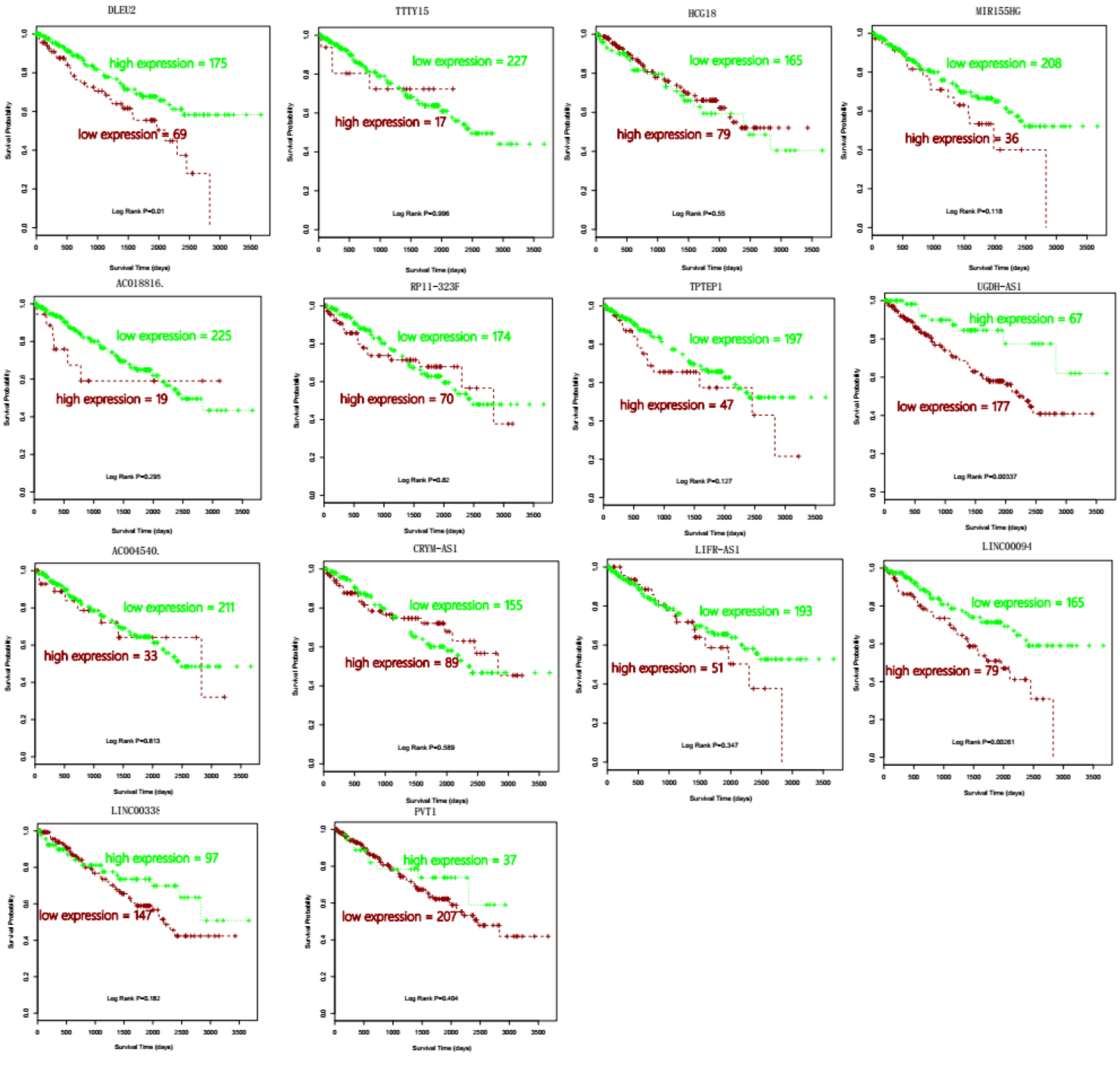


**Supplementary Figure S4: Kaplan-Meier survival analysis of two groups of patients for each hub with different clinical outcomes in KIRC data set.** Survival days are shown along the X axis. Overall survival rates are shown along the Y axis.

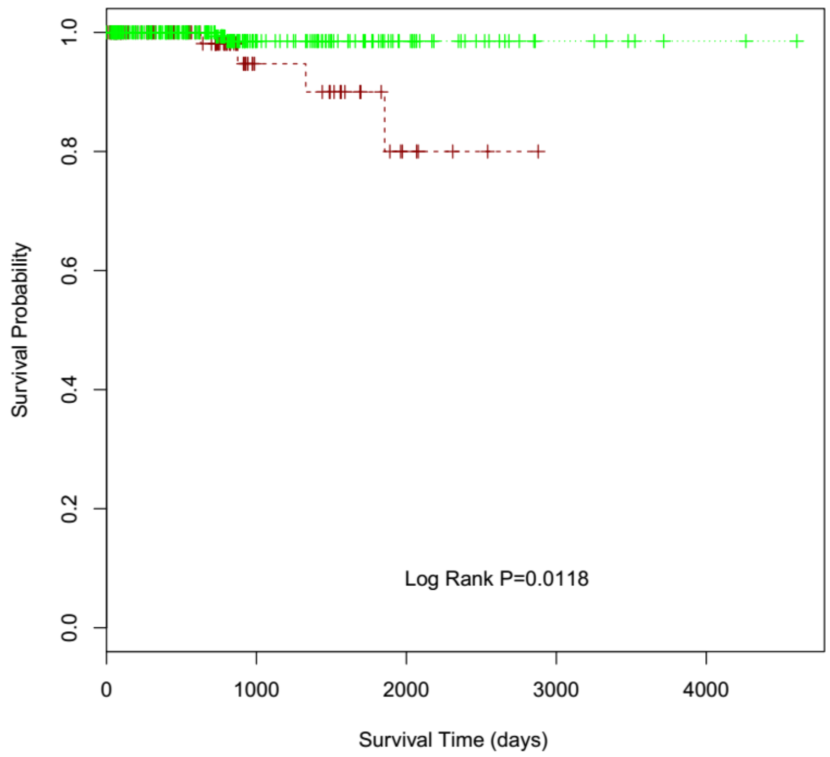


**Supplementary Figure S5: Kaplan-Meier survival analysis of two groups of patients based on expression quantity of hub lncRNAs in PRAD data set.** Survival days are shown along the X axis. Overall survival rates are shown along the Y axis.

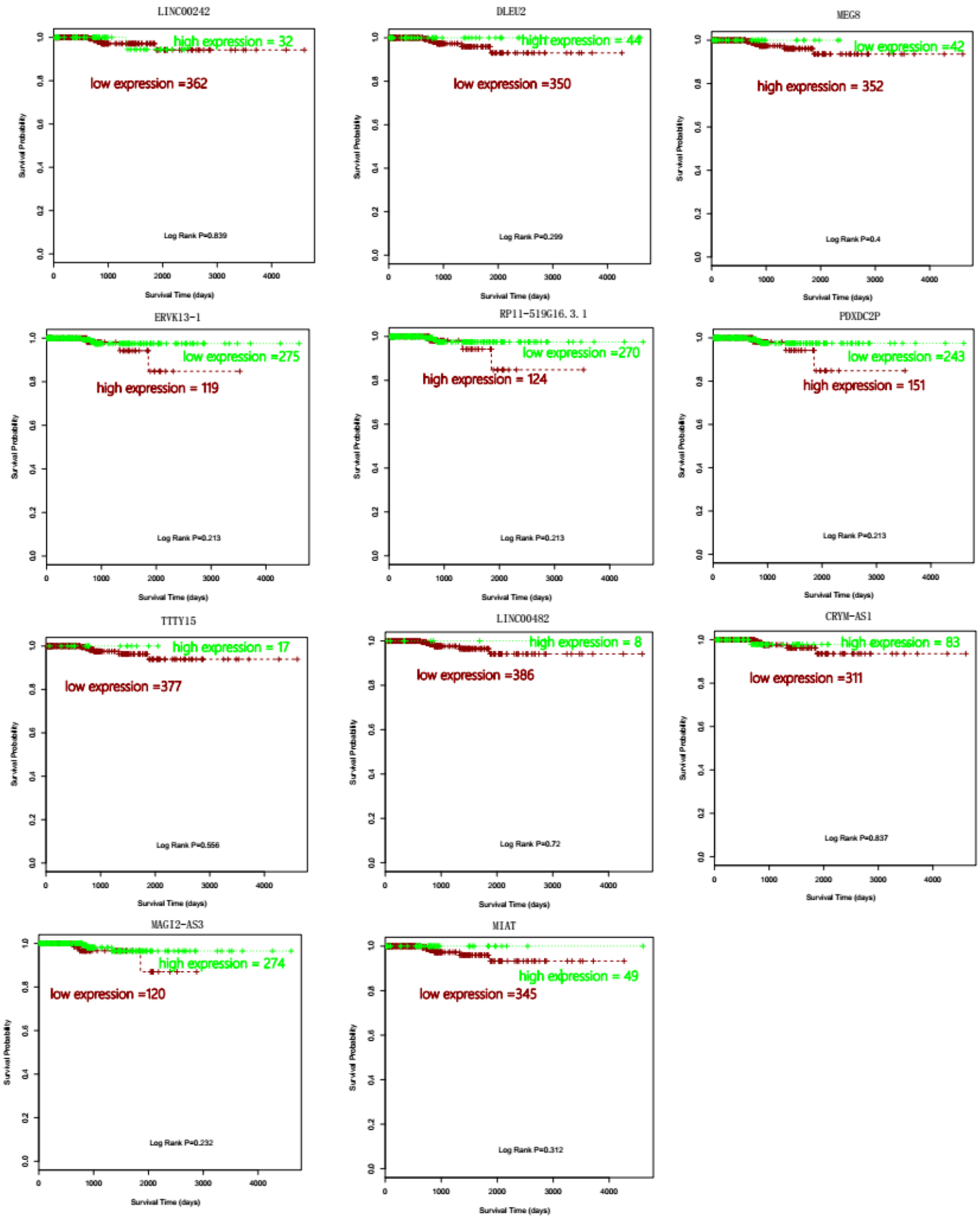


**Supplementary Figure S6: Kaplan-Meier survival analysis of two groups of patients for each hub with different clinical outcomes in PRAD data set.**

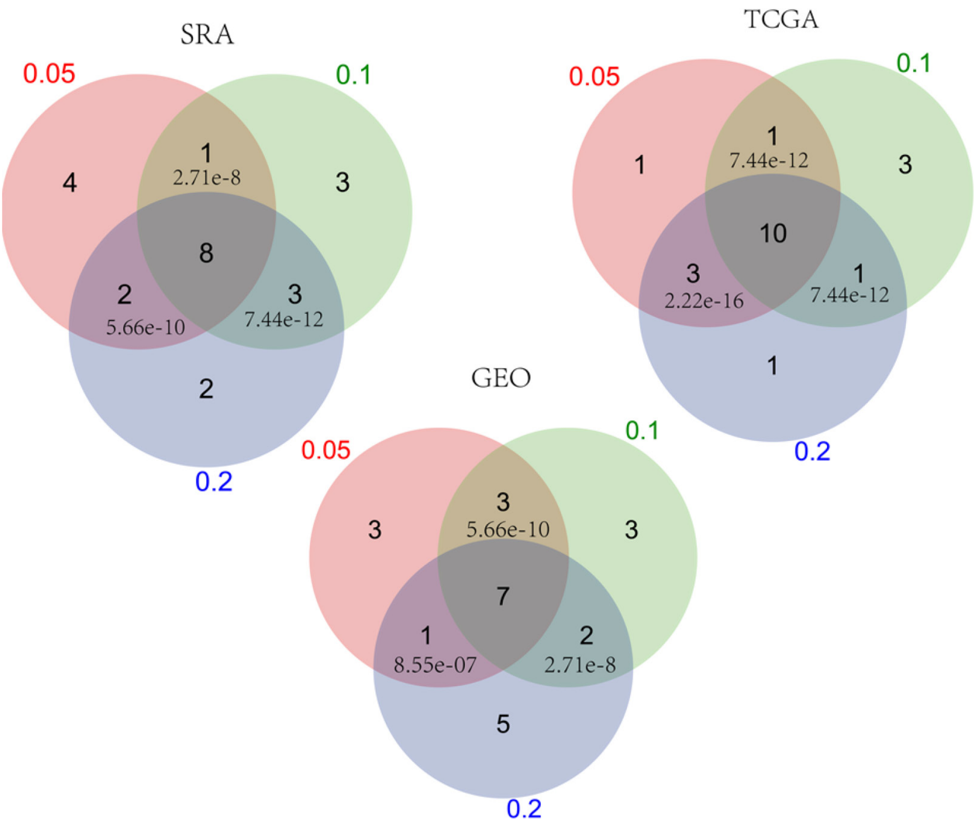


**Supplementary Figure S7: Reproducibility analysis.** Venn diagram depicts top 15 pathways identified by Subpathway-LNCE based on different *P* value(0.05, 0.1, 0.2) of differential genes in the same data sets for all three different data sources.
